# Rationale and design of the Clinical Evaluation of MAgnetic Resonance imaging in Coronary heart disease 2 trial (CE-MARC 2): A prospective, multi-centre, randomized controlled trial of diagnostic strategies for suspected coronary heart disease

**DOI:** 10.1186/1532-429X-18-S1-P75

**Published:** 2016-01-27

**Authors:** David P Ripley, Julia Brown, Colin C Everett, Petra Bijsterveld, Simon Walker, Mark Sculpher, Gerry P McCann, Colin Berry, Sven Plein, John P Greenwood

**Affiliations:** 1 grid.9909.90000000419368403Multidisciplinary Cardiovascular Research Centre (MCRC) & Leeds Institute of Cardiovascular and Metabolic Medicine, University Of Leeds, Leeds, UK; 2 grid.9909.90000000419368403Clinical Trials Research Unit, University of Leeds, Leeds, UK; 3 grid.5685.e0000000419369668Centre for Health Economics, University of York, York, UK; 4 grid.9918.90000000419368411Department of Cardiovascular Sciences, University of Leicester, Leicester, UK; 5 grid.8756.c000000012193314XBHF Glasgow Cardiovascular Research Centre, University of Glasgow, Glasgow, UK

## Background

A number of investigative strategies exist for the diagnosis of coronary heart disease (CHD). Despite the widespread availability of non-invasive imaging, invasive angiography is commonly used early in the diagnostic pathway. Consequently, approximately 60% of angiograms reveal no evidence of obstructive coronary disease. Reducing unnecessary angiography has potential financial savings and avoids exposing the patient to unnecessary risk. There are no large scale comparative effectiveness trials of the different diagnostic strategies recommended in international guidelines and none that have evaluated the safety and efficacy of cardiovascular magnetic resonance (CMR).

## Methods

CE-MARC 2 is a prospective, multi-centre, 3-arm parallel group, randomized controlled trial of patients with suspected CHD (pre-test likelihood 10-90%) requiring further investigation. 1200 patients will be randomized on a 2:2:1 basis to receive 3.0 Tesla CMR-guided care, single photon emission computed tomography (SPECT) guided care (according to ACC/AHA appropriate-use criteria) or National Institute for Health and Care Excellence guidelines-based management. The primary (efficacy) endpoint is the occurrence of unnecessary angiography as defined by a normal (>0.8) invasive fractional flow reserve. Safety of each strategy will be assessed by 3-year major adverse cardiovascular event rates. Cost effectiveness and health related quality of life (HRQoL) measures will be performed.

## Results

The CE-MARC 2 trial will provide comparative efficacy and safety evidence for three different strategies of investigating patient with suspected CHD, with the intension of reducing unnecessary invasive angiography rates.

## Conclusions

Evaluation of these management strategies has the potential to improve patient care, HRQoL and the cost effectiveness of CHD investigation.

**Figure 1:**
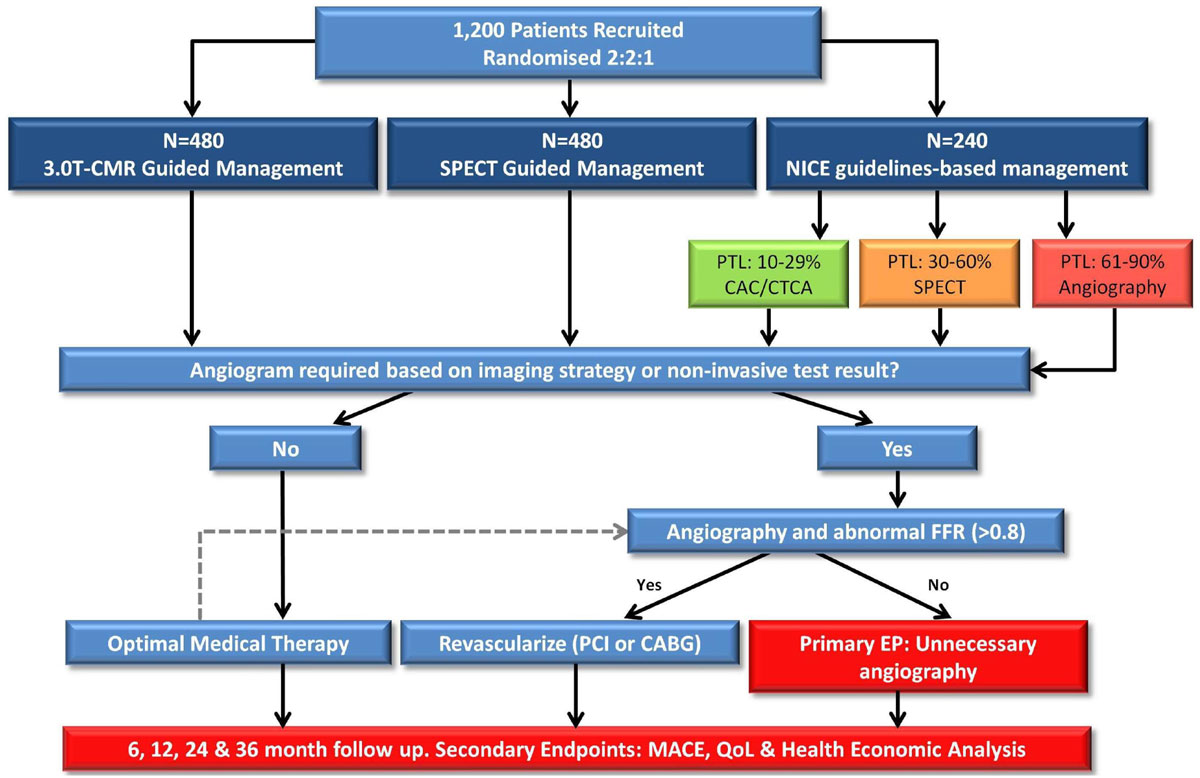
**CE-MARC 2 study flow diagram illustrating randomization, investigative strategy and study end-points**.

